# Nicotinamide mononucleotide attenuates brain injury after intracerebral hemorrhage by activating Nrf2/HO-1 signaling pathway

**DOI:** 10.1038/s41598-017-00851-z

**Published:** 2017-04-06

**Authors:** Chun-Chun Wei, Yuan-Yuan Kong, Guo-Qiang Li, Yun-Feng Guan, Pei Wang, Chao-Yu Miao

**Affiliations:** 1 Department of Pharmacology, Second Military Medical University, Shanghai, China; 2 Key Laboratory of Molecular Pharmacology and Drug Evaluation, Ministry of Education, Yantai University, Yantai, China; 3 Center of Stroke, Beijing Institute for Brain Disorders, Beijing, China

## Abstract

Replenishment of NAD^+^ has been shown to protect against brain disorders such as amyotrophic lateral sclerosis and ischemic stroke. However, whether this intervention has therapeutic effects in intracerebral hemorrhage (ICH) is unknown. In this study, we sought to determine the potential therapeutic value of replenishment of NAD^+^ in ICH. In a collagenase-induced ICH (cICH) mouse model, nicotinamide mononucleotide (NMN), a key intermediate of nicotinamide adenine dinucleotide (NAD^+^) biosynthesis, was administrated at 30 minutes post cICH from tail vein to replenish NAD^+^. NMN treatment did not decrease hematoma volume and hemoglobin content. However, NMN treatment significantly reduced brain edema, brain cell death, oxidative stress, neuroinflammation, intercellular adhesion molecule-1 expression, microglia activation and neutrophil infiltration in brain hemorrhagic area. Mechanistically, NMN enhanced the expression of two cytoprotective proteins: heme oxygenase 1 (HO-1) and nuclear factor-like 2 (Nrf2). Moreover, NMN increased the nuclear translocation of Nrf2 for its activation. Finally, a prolonged NMN treatment for 7 days markedly promoted the recovery of body weight and neurological function. These results demonstrate that NMN treats brain injury in ICH by suppressing neuroinflammation/oxidative stress. The activation of Nrf2/HO-1 signaling pathway may contribute to the neuroprotection of NMN in ICH.

## Introduction

Intracerebral hemorrhage (ICH) is a devastating type of stroke occurring when an abnormal blood vessel within the brain disrupts, allowing blood to leak inside the brain tissue. Although hemorrhagic strokes are less common, accounting less than 15% of all strokes cases, they are responsible for about half of all stroke deaths, and is associated with worse recovery than ischemic stroke both in world^1^. The primary brain injury induced by ICH, which is always considered to be hematoma-caused mechanical damage, takes place within several minutes to hours after the onset of bleeding. Secondary injury is resulted by the subsequent pathophysiological changes and the complex interaction between them. These pathophysiological changes following ICH include, but are not limited to, blood-brain barrier breakdown, hemoglobin-induced iron overload, excitotoxicity, neuroinflammation activation, triggered oxidative stress and neural cell death/apoptosis^2, 3^. Currently, large scale clinical trials have not reached a consensus on the benefit of surgical evacuation in treatment of ICH-induced primary injury^4^. Thus, targeting the secondary injury attracts great attentions for development of novel therapeutic strategies for ICH.

NAD^+^ is a well-known ubiquitous pyridine nucleotide that functions as an essential cofactor in mitochondrial oxidative phosphorylation. Classically, NAD^+^ is considered to be just a coenzyme which is essential for mitochondrial electron transfer chain^5^. However, overwhelming evidence in recent years has demonstrated that NAD^+^ not only acts as a coenzyme, but also participates in the transduction of numerous important intracellular signaling pathways to critically regulate numerous biological functions including cell death, metabolism, circadian rhythms, aging and immunity through regulating several NAD^+^-consuming proteins such as sirtuin family proteins and poly(ADP-ribose) polymerases^6–8^. As NAD^+^ depletion is a necessary event for neuronal death^9, 10^, supplement of NAD^+^ is neuroprotective through enhancing NAD^+^ pool. Our group has provided numerous evidence of the neuroprotection of NAD^+^^11–15^. Nicotinamide mononucleotide (NMN) is a particularly interesting chemical compound used to replenish NAD^+^. NMN is a key intermediate of nicotinamide adenine dinucleotide (NAD^+^) biosynthesis from nicotinamide, which is catalyzed by nicotinamide phosphoribosyltransferase (NAMPT) in mammals^16^. Recent evidence suggests that NMN treats obesity^17^, vascular aging^18^ and islet damage^19^. Besides, NMN has favorable effects in central nerve system (CNS) by raising brain mitochondrial respiratory deficits^20^, protecting β-amyloid oligomer-induced cognitive impairment^21^, delaying astrocyte-mediated motor neuron death^22^ and maintaining neural stem/progenitor cells^23^. We and other group also previously demonstrated that NMN protectes against cerebral ischemia-induced neural apoptosis and promotes neurogenesis after cerebral ischemia^13, 24, 25^. The potential therapeutic values of NMN in cerebral ischemic stroke have been discussed in detail in our previous review^26^. However, the effects of NMN in hemorrhagic stroke have not been examined yet.

In the present study, we conducted a straightforward study to determine whether replenishment of NAD^+^ by NMN, the key intermediate of NAD^+^ biosynthesis, is able to enhance intracerebral NAD^+^ pool and treat ICH-induced brain injury in animal model and, if so, to further explore the molecular mechanisms underlying the therapeutic action of NMN in ICH.

## Results

### NMN treatment protects against cICH-induced acute brain injury

**Figure 1:**
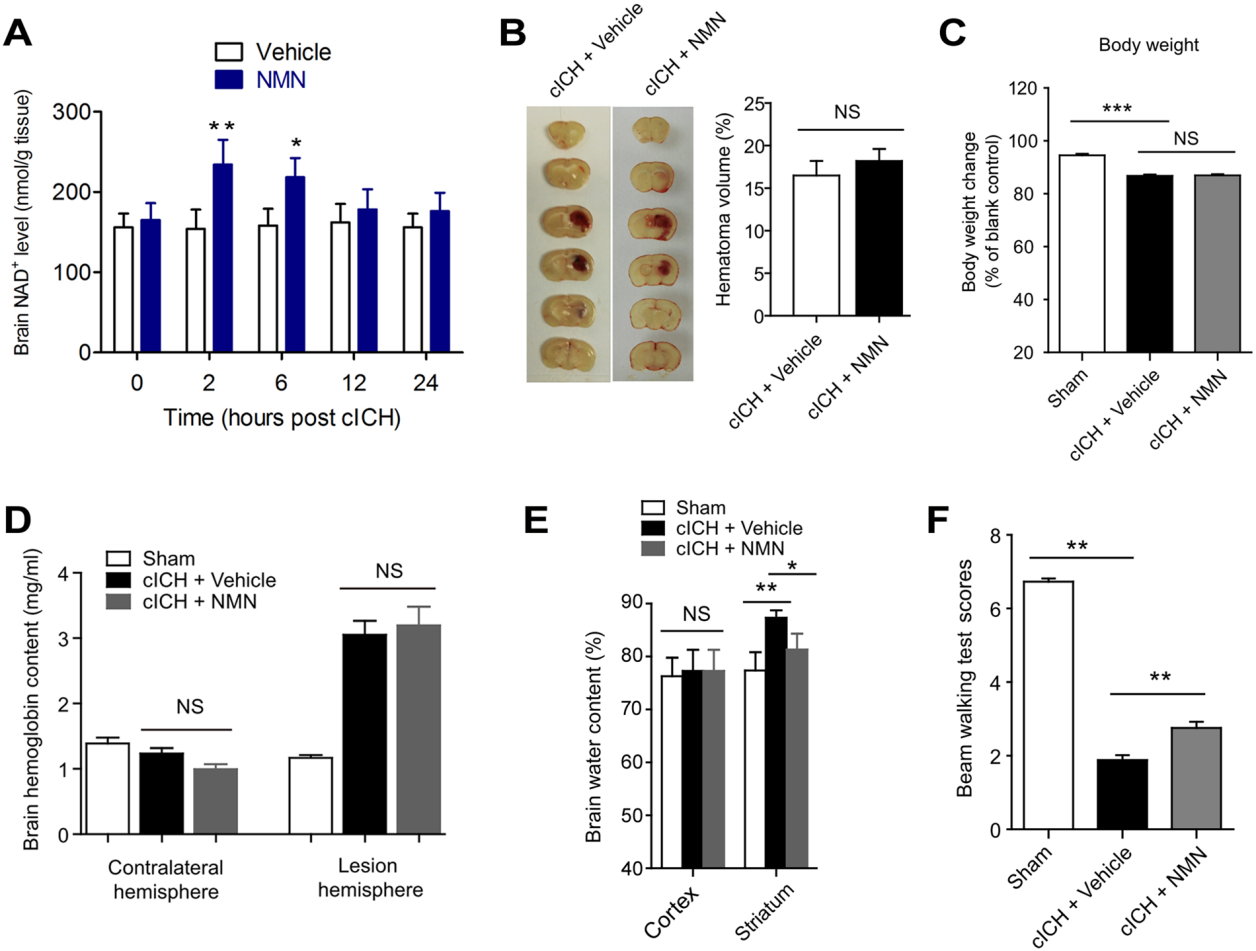
Nicotinamide mononucleotide (NMN) treatment protects against cICH-induced brain injury. (**A**) NAD^+^ level in brain tissue was measured at 2 hours after injection of NMN from tail vein. *P < 0.05 vs Vehicle; NS, no significance. N = 6 per group. (**B**) Brain hematoma volume at 24 hours after cICH. n = 8 per group. (**C**) Body weight at 24 hours after cICH. ***P < 0.001 vs Sham, n = 22, 60 and 63 in Sham, cICH + Vehicle and cICH + NMN groups respectively. (**D**) Hemoglobin content at 24 hours after cICH. n = 8 per group. (**E**) Brain water content at 24 hours after cICH in cortex and striatum. **P < 0.01 cICH + Vehicle vs Sham, *P < 0.05 cICH + NMN vs cICH + Vehicle, n = 8. (**F**) Neurological deficit was evaluated by beam walking test at 24 hours after cICH. **P < 0.01, n = 26, 69 and 73 in Sham, cICH + Vehicle and cICH + NMN groups respectively. NS, no significance.

Hematoma volume, brain hemoglobin content, body weight, brain water content and neurological function deficit were evaluated at 24 h after cICH. A single dose of NMN treatment given at 30 minutes (i.v.) post cICH increased intracerebral NAD^+^ concentrations at 2 and 6 hours post cICH (Fig. 1A). At 12 and 24 hours later, the NAD^+^ level returned to basal (Fig. 1A). NMN treatment did not affect the hematoma volume (Fig. 1B), decline of body weight (Fig. 1C) and brain hemoglobin content (Fig. 1D). Interestingly, the induction of edema in striatum (water content) by cICH was slightly but significantly reduced by NMN treatment (Fig. 1E). Beam walking test demonstrated that NMN improved the neurological function at 24 hours after cICH (Fig. 1F). These data suggest that NMN is unable to reduce hematoma volume, but it alleviates ICH-induced brain injury.

### NMN treatment reduces brain cell death and oxidative stress in mouse cICH model

**Figure 2:**
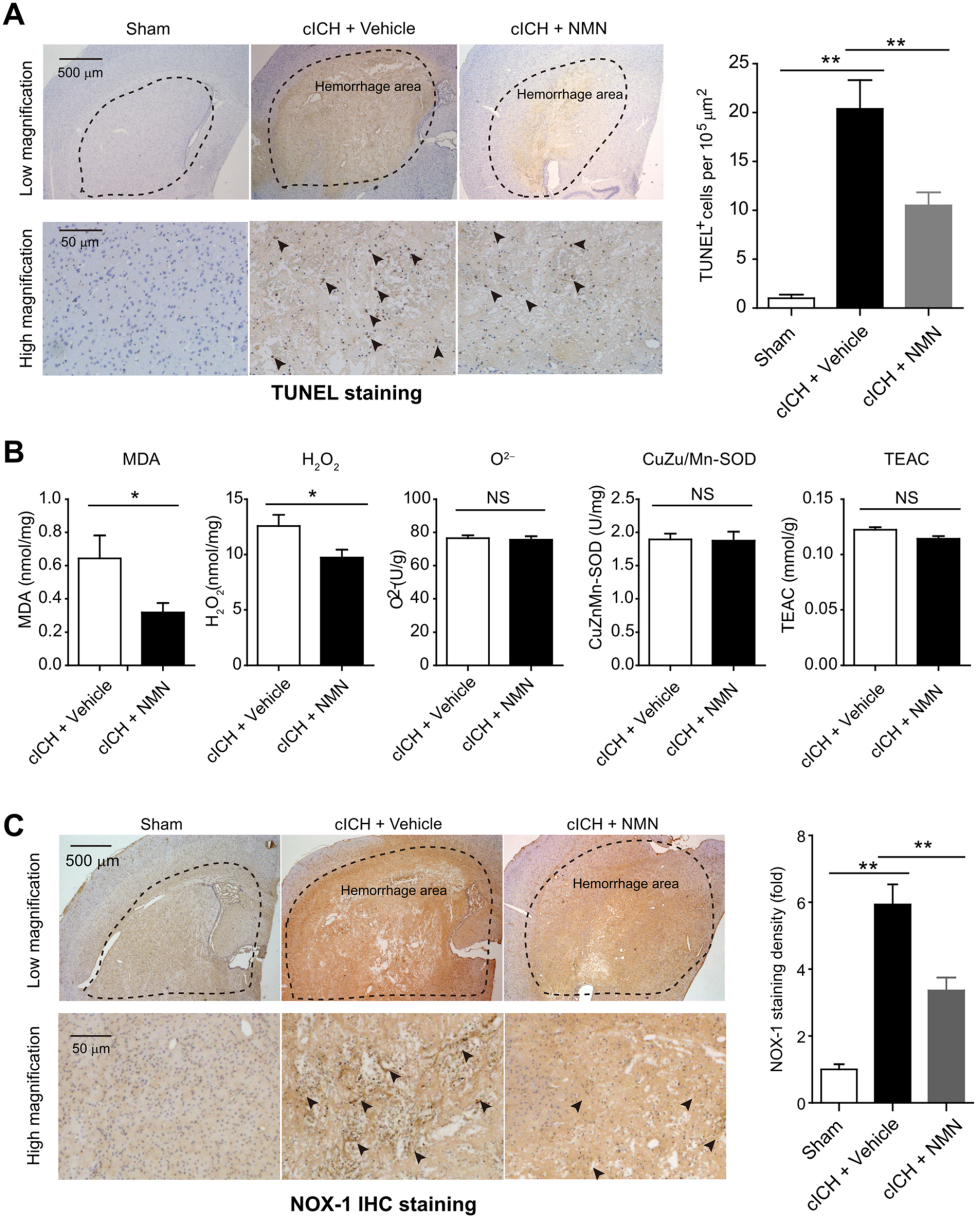
Effects of NMN on brain cell death and oxidative stress in mouse cICH model. (**A**) Representative images and quantitative analysis of TUNEL staining at 24 hours post cICH. **P < 0.01, n = 5 per group. (**B**) Oxidative parameters in hemorrhagic brain tissue, including MDA, H_2_O_2_, O^2−^, CuZn/Mn-SOD, total-antioxidant activity, were determined at 24 hours post cICH. *P < 0.05, n = 6 per group. (**C**) Representative images and quantitative analysis of NOX-1 immunohistochemistry staining at 24 hours post cICH. **P < 0.01, n = 5 per group.

Next, we studied the influence of NMN treatment on brain cell death and oxidative stress in mouse cICH model. Low magnification images in TUNEL assay (upper row) showed that there was a significant TUNEL-positive staining in deep cortex/striatum area (hemorrhage area) in mouse cICH model (Fig. 2A). Under high magnification (lower row), we found that the number of TUNEL-positive cells in hemorrhage area in NMN-treated mice was significantly lower than that in vehicle-treated mice (Fig. 2A).

NMN treatment significantly reduced MDA level and H_2_O_2_ level in hemorrhage area (Fig. 2B). However, NMN treatment did not alter the levels of O^2−^, CuZu/Mn-SOD activity and total anti-oxidant activity (Fig. 2B). NOX-1 is a member of the NADPH oxidase family that critically contributes to the generation of intracellular oxidative stress^27, 28^. Immunohistochemistry staining showed that the NOX-1-positive cells were mainly located at hemorrhage area (Fig. 2C). We also found that the NOX-1 immunohistochemistry staining density in NMN-treated mice was remarkably lower than that in vehicle-treated mice (Fig. 2C). These results demonstrate that NMN treatment reduces cell death and oxidative stress in mouse cICH model.

### NMN treatment suppresses neuroinflammation in mouse cICH model

**Figure 3:**
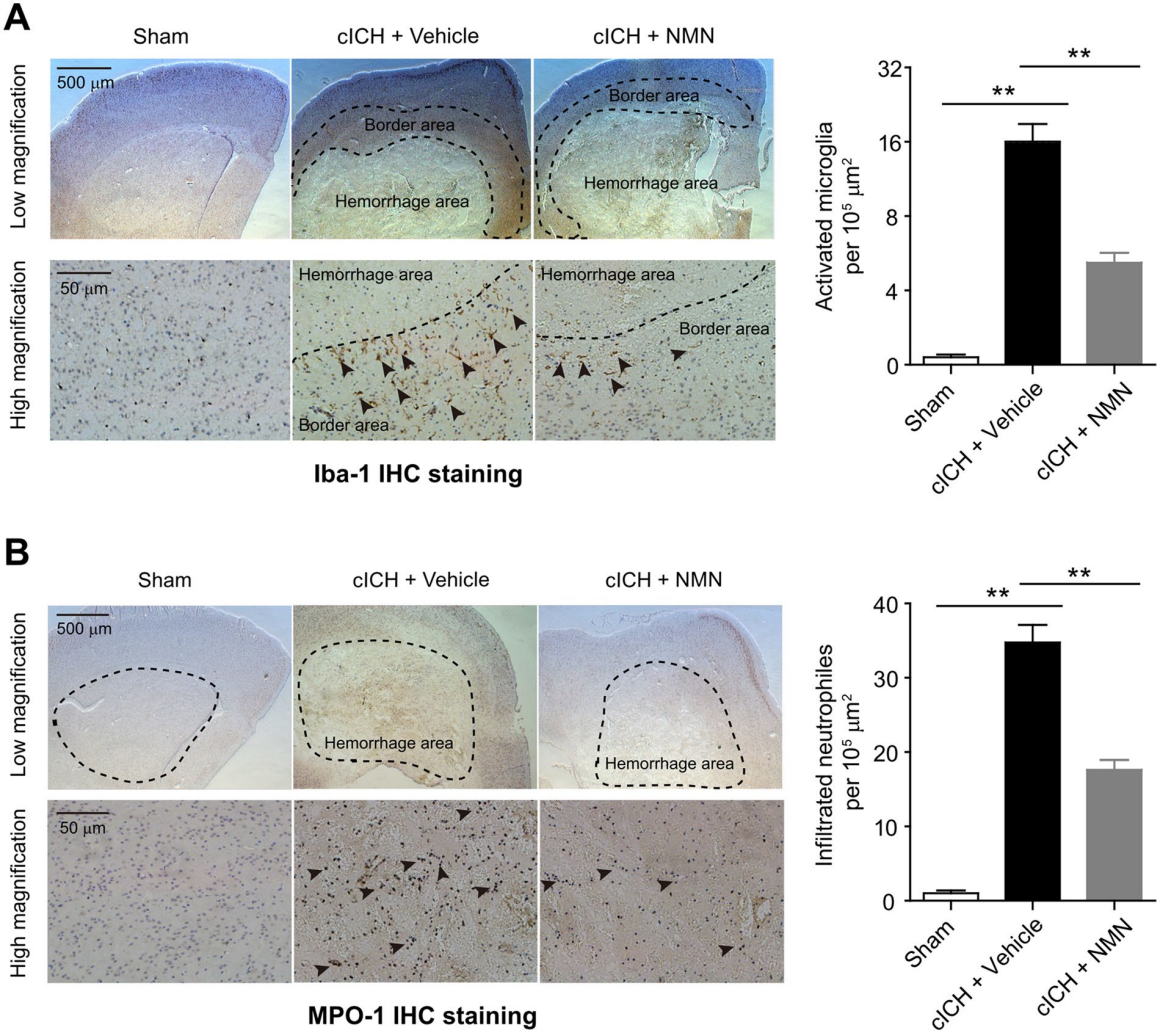
Effects of NMN on microglia activation and neutrophil infiltration in mouse cICH model. (**A**) Representative images and quantitative analysis of Iba-1 (microglia marker) immunohistochemistry staining at 24 hours post cICH. **P < 0.01, n = 5 per group. (**B**) Representative images and quantitative analysis of MPO-1 (neutrophil marker) immunohistochemistry staining at 24 hours post cICH. **P < 0.01, n = 5 per group.

To assess the effect of NMN on microglia activation and neutrophil infiltration, we detected Iba-1 and MPO-1 protein levels in brain tissue by immunohistochemistry staining at 24 hours following cICH respectively. As shown in Fig. 3A, the Iba-1-positive microglia (activated) was mainly located at the border area surrounding the hemorrhagic core area but not the hemorrhagic core area itself. NMN treatment significantly suppressed the microglia activation (Fig. 3A). The neutrophil infiltration, which was referred by MPO-1 protein level, was also markedly induced by cICH but inhibited by NMN treatment (Fig. 3B).

### NMN treatment inhibits the pro-inflammatory factors levels in mouse cICH model

**Figure 4:**
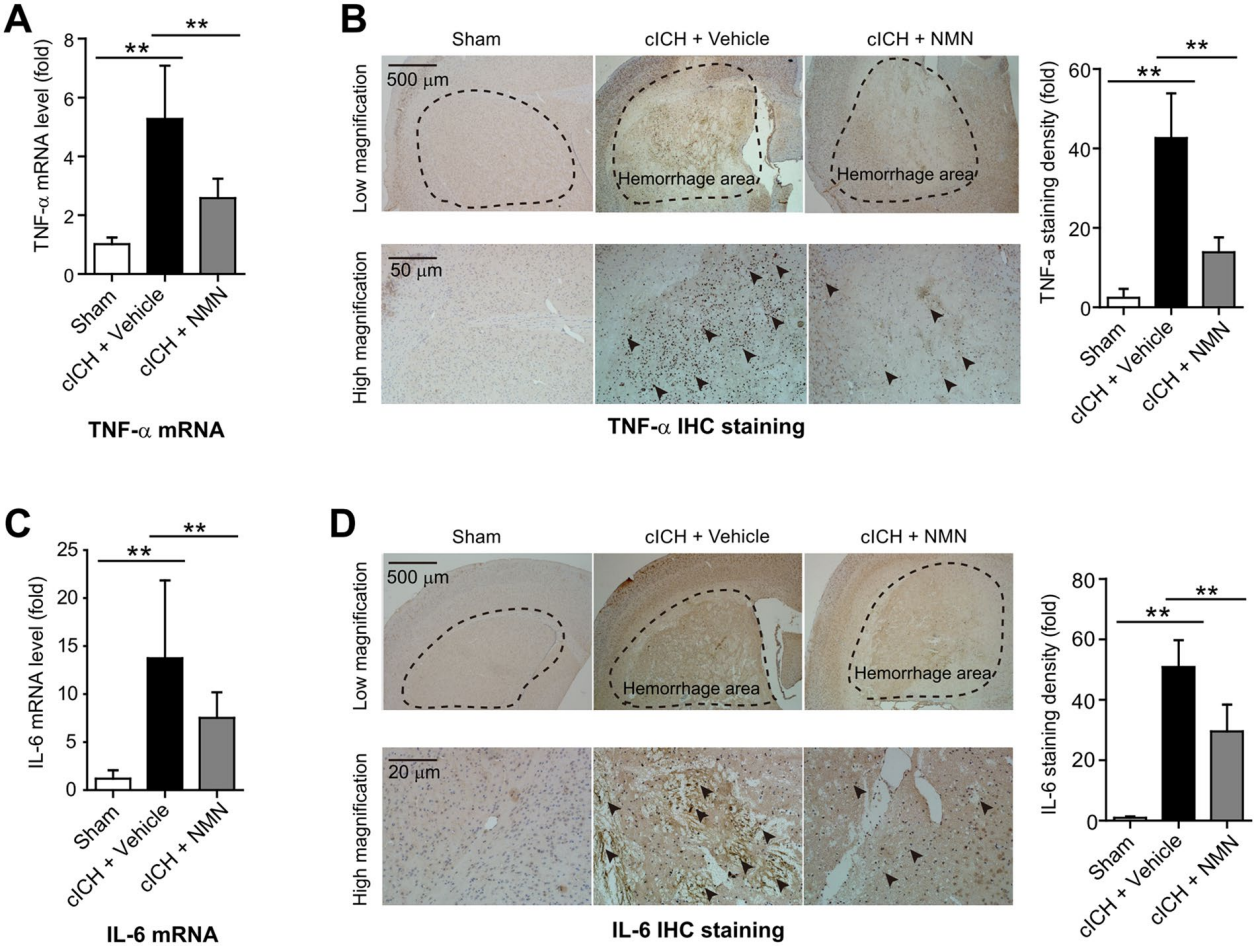
Effects of NMN on expression of TNF-α and IL-6 in mouse cICH model. (**A**) The mRNA level of TNF-α in hemorrhagic brain tissue was evaluated at 24 hours post cICH by real-time PCR. **P < 0.01. (**B**) The protein level of TNF-α in hemorrhagic brain tissue was evaluated at 24 hours post cICH by immunohistochemistry staining. **P < 0.01. (**C**) The mRNA level of IL-6 in hemorrhagic brain tissue was evaluated at 24 hours post cICH by real-time PCR. **P < 0.01. (**D**) The protein level of IL-6 in hemorrhagic brain tissue was evaluated at 24 hours post cICH by immunohistochemistry staining. **P < 0.01. n = 3, 10 and 9 in Sham, cICH + Vehicle and cICH + NMN groups respectively for (**A–D**).

We assessed the expression of inflammatory-associated factors^29^ to evaluate the neuroinflammation activation after cICH. TNF-α mRNA level was induced by ~5-fold in cICH brain tissue, which was significantly repressed by NMN treatment (Fig. 4A). TNF-α immunohistochemistry staining confirmed this result (Fig. 4B). Moreover, the increases of IL-6 mRNA (Fig. 4C) and protein (Fig. 4D) expression were also triggered by cICH and compromised by NMN treatment. We also tested the expression of one other pro-inflammatory factor (IL-1β) and two anti-inflammatory factors (IL-10 and IL-4). ICH induced brain IL-1β and IL-10 mRNA levels, and reduced IL-4 mRNA level (Supplemental Fig. 1). However, NMN treatment did not affect the mRNA changes of IL-1β, IL-10 and IL-4 (Supplemental Fig. 1).

### NMN treatment reduces ICAM-1 but not VCAM-1 protein expression in mouse cICH model

**Figure 5:**
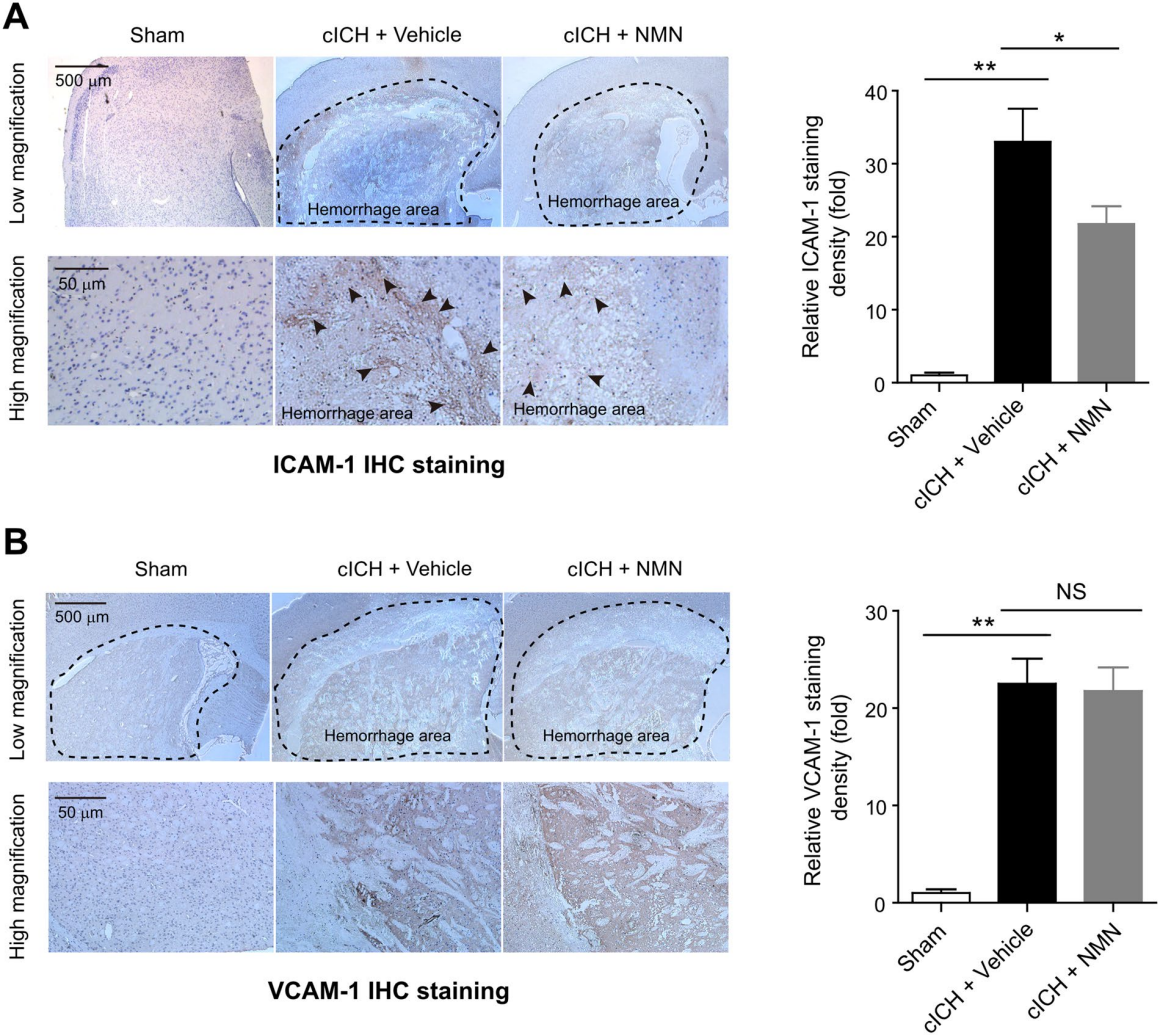
Effects of NMN on the expression of ICAM-1 and VCAM-1 in mouse cICH model. (**A**) Representative immunohistochemistry staining images and quantitative analysis of ICAM-1 at 24 hours post cICH; **P < 0.01 cICH + Vehicle vs Sham, *P < 0.05 cICH + NMN vs Cich + Vehicle, n = 5 per group. (**B**) Representative immunohistochemistry staining images and quantitative analysis of VCAM-1 at 24 hours post cICH; **P < 0.01 cICH + Vehicle vs Sham, *P < 0.05 cICH + NMN vs cICH + Vehicle, n = 5 per group. IHC, immunohistochemistry.

Chemokines and adhesion molecules are crucial for the procedure of neuroinflammation activation post ICH and monocyte trafficking across the vessel wall^30–32^. Immunohistochemistry staining showed that the ICAM-1- and VCAM-1-positive areas were mainly located at the hemorrhagic lesion area in mouse cICH model (Fig. 5A,B). NMN treatment successfully decreased the immunohistochemistry density of ICAM-1 (Fig. 5A); however, it did not reduce the expression of VCAM-1 (Fig. 5B).

### NMN treatment activates Nrf2/HO-1 signaling pathway in mouse cICH model

**Figure 6:**
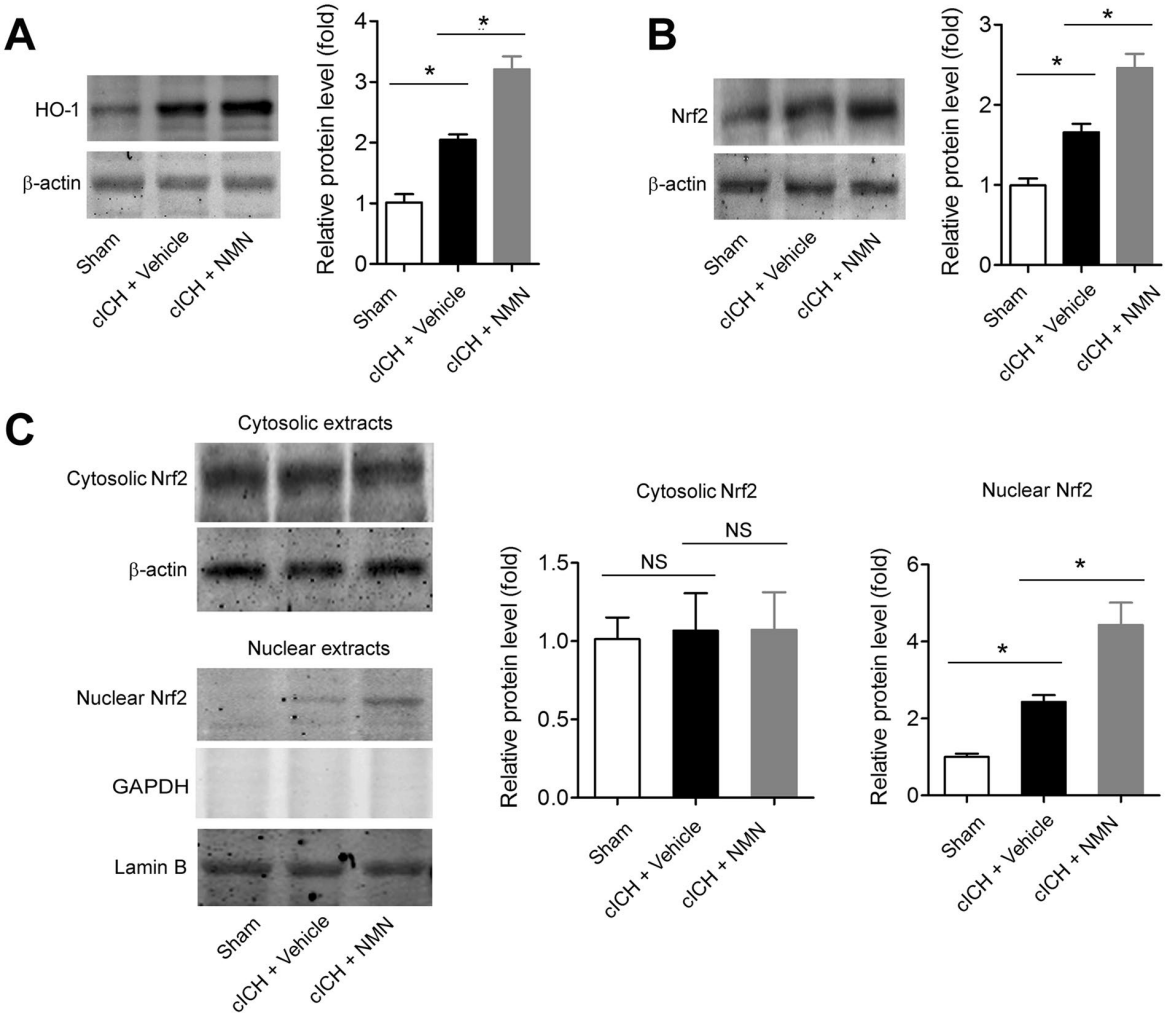
Involvement of Nrf2/HO-1 signaling pathway in therapeutic effects of NMN. (**A**) Representative images and quantitative analysis of HO-1 protein in peri-hemorrhagic brain tissues at 24 hours after cICH. (**B**) Representative images and quantitative analysis of Nrf2 protein in peri-hemorrhagic brain tissues at 24 hours after cICH. (**C**) Representative images and quantitative analysis of Nrf2 protein distribution in cytosolic and nuclear extracts of brain peri-hemorrhagic tissues at 24 hours after cICH. *P < 0.01, n = 5 per group. NS, no significance.

To investigate the molecular mechanisms behind the neuroprotection of NMN against ICH-induced brain injury, we studied the protein expression of HO-1 and Nrf2 in the mouse cICH model. HO-1 was significantly upregulated in brain tissues from cICH-treated mice (Fig. 6A). NMN further enhanced the HO-1 expression (Fig. 6A). The protein expression of Nrf2, which is an upstream regulator of HO-1^33, 34^, was also studied. Similarly, Nrf2 expression was upregulated in ICH condition and further increased by NMN treatment (Fig. 6B). We next investigated the nuclear translocation of Nrf2. Under normal condition, Nrf2 is mainly located in cytosol extraction, but not in nuclear extraction (lower panel, Fig. 6C). Upon ICH stress or NMN treatment, the cytosolic Nrf2 expression was not altered (upper panel, Fig. 6C). However, the nuclear Nrf2 was significantly increased under ICH condition and further enhanced by NMN treatment (lower panel, Fig. 6C). These results suggest that Nrf2/HO-1 signaling pathway is involved in the therapeutic profile of NMN against hemorrhagic stroke.

### NMN treatment reduces brain injury at 3 days post cICH in mouse model

**Figure 7:**
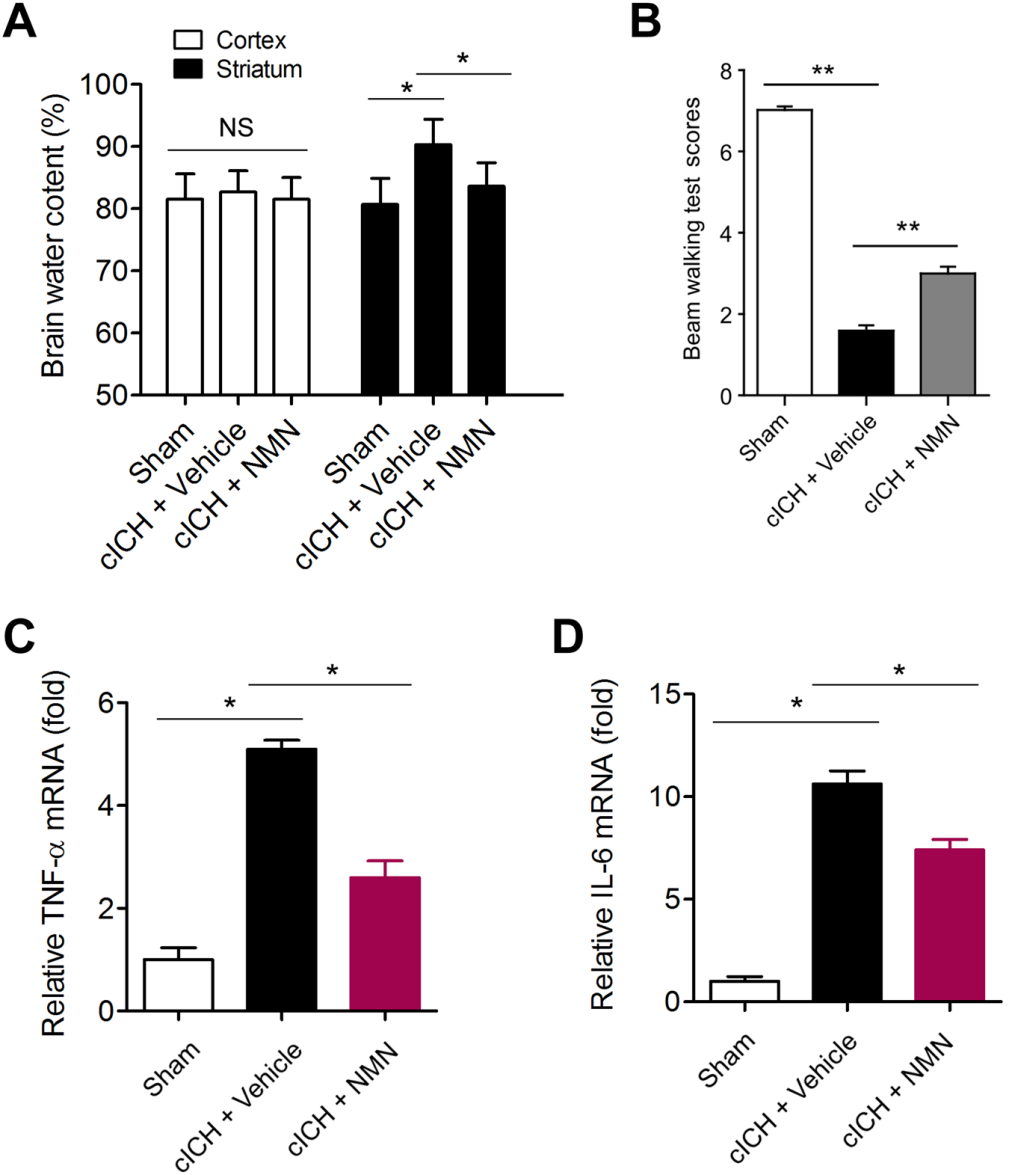
Effects of NMN on brain injury at 3 days post cICH in mouse model. (**A**) Brain water content at 3 days after cICH in cortex and striatum. *P < 0.05 cICH + Vehicle vs Sham, *P < 0.05 cICH + NMN vs cICH + Vehicle, n = 8. (**B**) Neurological deficit was evaluated by beam walking test at 3 days after cICH. *P < 0.05, n = 10 per group. (**C**,**D**) The mRNA level of TNF-α (**C**) and IL-6 (**D**) in hemorrhagic brain tissue was evaluated at 3 days after cICH by real-time PCR. **P < 0.05. n = 6 per group.

As the neuroinflammation and brain injury always reach the peak at 3 days after ICH, we examined brain injury and pro-inflammatory factors at this timepoint. As shown in Fig. 7A, NMN administration (300 mg/kg, i.p., 30 minutes after ICH) reduced brain edema at 3 days post cICH. NMN administration also rescued neurological deficit at 3 days post cICH (Fig. 7B). In addition, the TNF-α and IL-6 mRNA levels were remarkably decreased by NMN administration (Fig. 7C,D).

### A prolonged NMN treatment promotes survival in sub-acute phase in mouse cICH model

**Figure 8:**
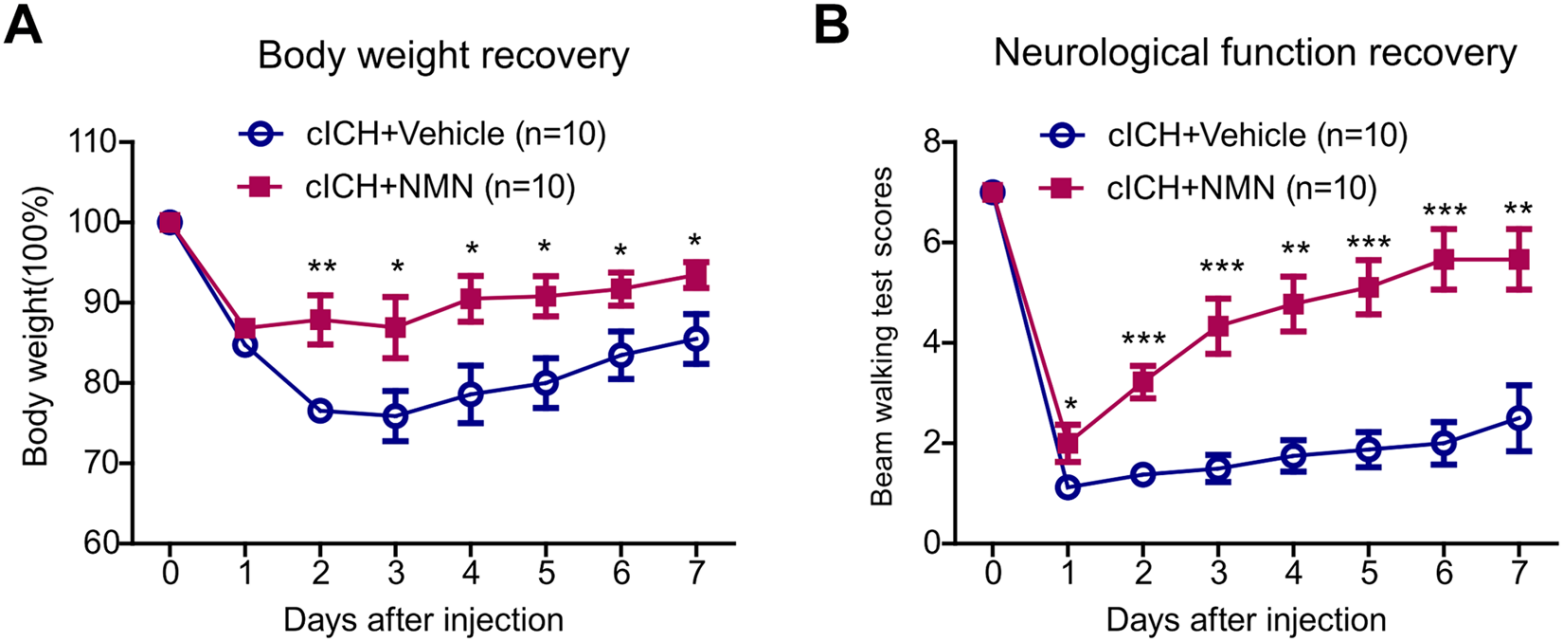
Effects of a 7-day treatment of NMN on body weight and neurological function recovery in mouse cICH model. Mice were treated with saline (control) or NMN (first dose at 30 minutes post cICH from tail vein [300 mg/kg] and subsequent dose by intraperitoneal injection for 7 days post cICH [300 mg/kg]). (**A**) Body weight curve. (**B**) Neurological score curve. n = 10 per group. *P < 0.05, **P < 0.01, ***P < 0.001 cICH + NMN vs cICH + vehicle.

Since all the above-mentioned experiments were conducted in mice in acute phase (~24 hours after cICH), we further determined the possible beneficial action of NMN in mice in sub-acute phase. NMN was administrated every day during the first week post cICH. The decline of body weight during the first week post cICH was partly reversed by prolonged NMN treatment (Fig. 8A). In line with this result, the neurological function in NMN-treated mice was recovered faster compared with vehicle-treated mice during the first week post cICH (Fig. 8B).

## Discussion

This study is the first to test the therapeutic potential of NMN against ICH-induced brain injury. Previously, we reported that the compound NMN conferred a pronounced neuroprotection in ischemic stroke^35^, and promoted neurogenesis post ischemic stroke^24^. In the present study, we found NMN was failed to reduce hematoma volume and hemoglobin content. However, a bolus injection of NMN at 30 minutes post cICH significantly improved neurological function after cICH. Importantly, this intervention reduced oxidative stress, depressed pro-inflammatory factors, decreased microglia activation/neutrophil infiltration, and attenuated chemokine ICAM-1 expression. Moreover, we further tried a 7-day treatment of NMN in mice with cICH. Continued treatment of NMN for one week remarkably rescued the body weight decline and the neurological deficit caused by cICH as we expect. Finally, we found that NMN treatment upregulated Nrf2 and HP-1 protein expression, and promoted Nrf2 nuclear translocation for its transactivation. Based on these results, we propose that NMN has suppressive effects on post-ICH neuroinflammation to attenuate the secondary neurological injury, although this compound has no effect on the primary injury during ICH.

NMN is a small-sized precursor of NAD^+^ that represents a classic coenzyme in well-established cellular redox reaction for producing energy (adenosine triphosphate, ATP). Recently, the understanding on NAD^+^ has been expanded as the NAD^+^ biochemistry is implicated in a broader range of fundamental intracellular biological functions^6, 7^. It should be noted that the concept “NAD^+^ pool” implies that the concentration of NAD^+^ is controlled by both of its biosynthesis and cleavage. Using a fluorimetric NMN-based detection technology, Formentini *et al*. analyzed the kinetics of NAD^+^ recycling in Hela and U937 lymphoma cells, and found that NMN is highly enriched in mitochondria, suggesting that the intramitochondrial NAD^+^ synthesis is largely dependent on NMN^36^. NMN supplementation was shown to be an effective therapy protecting against redox-induced cell death^37, 38^, high-fat diet induced obesity^17^, pro-inflammatory cytokine-mediated islet function^19^, β-amyloid oligomer-induced cognitive impairment^21^, vascular dysfunction and aging^18^, and astrocyte-mediated motor neuron death in amyotrophic lateral sclerosis^22^. All these features of NMN were attributed to that it is a substrate for NAD^+^ biosynthesis. In this study, we showed that administration of NMN from tail vein enhanced intracerebral NAD^+^ levels in mouse cICH model. This result is in line with our previous results showing NMN injection from peripheral routes was sufficient to increase intracerebral NAD^+^ in murine ischemic stroke model^24^. Stein *et al*. also reported that the enhancement of NAD^+^ biosynthesis in the brain after a single NMN injection (500 mg/kg) could last for at least 6 hours^23^. Moreover, Yoon *et al*. demonstrated that hypothalamic nucleus showed 1.5- to 3.5-fold increases in NAD^+^ level after NMN injection^39^. Thus, administration of NMN appears to be a quick and efficient tool to raise intracerebral NAD^+^ level and thereby promote NAD^+^-dependent multiple biological functions.

In our study, the hemoglobin content after ICH can’t be rescued by NMN; alternatively, NMN inhibited the neuroinflammation and oxidative stress post ICH. The secondary damage is partly due to the toxic effects of hemin, a breakdown product of hemoglobin. Hemin is toxic to the cells by causing apoptosis and inducing local extensive inflammation/oxidative stress. So our data suggest that the pathological responses to hemin may be affected by NMN, and we turned to investigate the effects of NMN on HO-1 and Nrf2. HO-1 is a ubiquitous enzyme that oxidatively cleaves heme, a pro-oxidant, to produce biliverdin and carbon monoxide. HO-1 can be induced by hemin. The upregulation of HO-1 protein expression in brain tissues by NMN is apparently in line with the results on the inhibitory action of NMN to neuroinflammation and oxidative stress. Further, we found that NMN significantly increased the nuclear Nrf2 protein expression, but did not change cytosolic Nrf2 protein expression. As only the nuclear Nrf2 can promote HO-1 gene transcription to oppose apoptosis and necrosis^33, 34, 40^, the enhanced Nrf2 nuclear translocation by NMN indicates that the enhanced nuclear Nrf2, but not cytosolic Nrf2, may contribute to the neuroprotection of NMN against brain injury in ICH.

Researchers have not reached a consensus on the relationship between intracellular NAD+ and inflammation. Intracellular NAD+ level was shown to regulate TNF-α synthesis in macrophages^41^. Esposito *et al*. reported that blockade of NAD+ biosynthesis using NAMPT inhibitor FK866 reversed the damage in spinal cord injury^42^. Bruzzone *et al*. also found that catastrophic NAD+ depletion in activated T lymphocytes through NAMPT inhibitor FK866 reduced demyelination and disability in experimental autoimmune encephalomyelitis. However, there are also investigations showing the beneficial effect of NAD+ in neuroinflammation. For example, Tullius *et al*. demonstrated that NAD+ administration blocked neuroinflammation in experimental autoimmune encephalomyelitis (EAE) by targeting CD4^+^ T-cell differentiation^43^. Audrito *et al*. reported that enhancement of NAD+ promotes M2 macrophage polarization to suppress inflammation^44^. Our study is the first to report a correlation between the post-ICH neuroinflammation and the NAD+ pool. Previous results from our lab^13–15^ have revealed that NAMPT, the enzyme for NMN production, is neuroprotective in ischemic stroke. In such condition, NMN may also protect the neural cells against hemorrhage-induced injury, which would trigger lower intensity oxidative stress and neuroinflammation. According to our results in the present study, the activated neuroinflammation, as well as the oxidative stress, neural apoptosis and ICAM-1 expression in the mouse cICH model, were inhibited by administration of NMN. Niacin, a natural precursor of NAD+, inhibited the fat accumulation, oxidative stress and inflammation in hepatocytes under high-fat diet^45^. These results further support the neuroprotection of NMN/NAD+. Nevertheless, more evidence may be needed to clarify the role of NMN/NAD+ in post-ICH neuroinflammation.

In our study, data from mice are from the cICH model. In fact, two rodent models of ICH are most commonly used: injection of the enzyme collagenase (cICH) and injection of autologous blood (bICH). According to a comparative study, there seemed no difference in lesion size between models. There are greater mass effect and early mortality in bICH model, whereas cICH produces greater edema, inflammation, and cell death, loss of cortical connections and secondary shrinkage of the striatum^46^. Although our data demonstrates the therapeutic value of NMN against ICH-induced brain injury, further studies may be required to confirm if there is same action of NMN in bICH model.

In conclusion, our results demonstrate that NMN protects against ICH-induced brain injury via inhibiting neuroinflammation in mice, and highlight that this strategy may shed a light on the future treatment for ICH.

## Methods

### Animals

Male 8-week-old CD1 mice (22–30 g) were purchased from Sino-British SIPPR/BK Lab Animal Ltd (Shanghai, China) and housed with free access to chow and water. Animal use procedures were approved by the Laboratory Animal Care and Use Committee of the Second Military Medical University, China. All experiments were performed in accordance with relevant guidelines and regulations.

### Randomization and Blinding

Mice in the present study were divided into different groups and selected for all experiments randomly by laboratory technicians. During the experiment and data analysis, single-blind study design was applied.

### Mouse cICH model

Experimental cICH model in mice was induced by intrastriatal injection of collagenase as described previously^47^. Briefly, mice were anesthetized with 10% chloral hydrate (500 mg/kg, intraperitoneal injection) and positioned prone in a stereotaxic head frame (Stoelting, Wood Dale, IL). The calvarium was exposed by a midline scalp incision from the nasion to the superior nuchal line to retract the skin. A burr hole (0.2 mm posterior to bregma and 2.3 mm to the right of the midline) was made with a drill (Fine Scientific Tools, Foster City, CA). A 26-G needle on a Hamilton syringe was inserted with stereotaxic guidance 3.5 mm into the deep cortex/basal ganglia. The collagenase (0.05 units in 1 µl saline; Sigma, St Louis, MO) in the syringe was infused into the brain at a rate of 0.2 μl/min over 5 minutes. Saline with same volume was injected in Sham group. The needle was left in place for an additional 7 minutes after injection to prevent the possible leakage of collagenase solution. The craniotomy was then sealed with bone wax, and the scalp was closed with sutures. Body temperature was maintained at 37 °C by a heating pad throughout the procedure. After waking up, the mice were given free access to food and water. The mice without neurological deficit or the dead mice (~20–30% mortality) were excluded from the following analysis.

### NMN treatment

The compound NMN was purchased from Bontac-Biotech Synthesis Corp. (Shenzhen, China). The purity of NMN is >98%, which was confirmed by high performance liquid chromatography analysis. For acute NMN treatment, a single dose of NMN (300 mg/kg) dissolved in saline was given at 30 minutes post cICH from tail vein. This dosage was chosen according to previous results from us^24, 35^ and other groups^17, 18^. The same volume of saline was injected as vehicle control. At 24 hours after treatment, all the dead mice were excluded. And the live mice were killed using cervical dislocation to determine the effects of NMN.

In another set of experiment with one-week period, mice were administrated with the first dose of NMN (300 mg/kg, i.v.) at 30 minutes post cICH via tail vein. After waking up, the mice were maintained in cages. From the second day, they were injected with NMN (300 mg/kg/d, i.p.) or vehicle (saline) for 7 days. The body weight and neurological function was assessed every day to investigate the effects of NMN on post-ICH recovery.

### Neurological deficit determination

Neurological deficit was evaluated by beam-walking test by a blinded investigator as previously described with modification^48^. Briefly, mouse was placed on a beam (1.2 m long, 1.5 cm wide, and 50 cm high), and usage of hindlimb during crossing the beam was analyzed on the basis of an eight-point scale as well as a fault rate. A score of 0 was given when mouse could not balance on the beam (<5 seconds); 1 was given when mouse remained on the beam for >5 seconds but could not cross the beam; 2 was given when mouse could balance on the beam but not traverse it; 3 was given when mouse traversed the beam with the affected limb extended and not reaching the surface of the beam, or when mouse made a turn on the beam; 4 was given when mouse traversed the beam with 100% footslips; 5 was given when mouse traversed the beam with >50% but <100% footslips; 6 was given when mouse traversed the beam with <50% footslips; 7 was given when mouse traversed the beam with two or less footslips. Performance on each day was expressed as an average score of three trials. Fault rate was presented as an average of three trials.

### Brain water content

Brain water content, a measure of brain edema due to BBB breakdown post-cICH, was determined as described previously^47^. Brains were removed and three parts (ipsilateral cortex and ipsilateral basal ganglia) were isolated immediately. Tissue samples were weighed on an electronic analytical balance (model AE 100; Mettler Instrument Co., Columbus, OH) to the nearest 0.1 mg to obtain the wet weight. The tissue was then dried at 100 °C for 48 h to obtain dry weight. The water content of brain tissue = [(wet weight) − (dry weight)]/(wet weight) × 100%.

### Brain hematoma size

Briefly, mice were euthanized under deep anesthesia. Brains were removed immediately and put in −20 °C for 1 hour. Then, the brain was cut into six sections. Brain slices were mounted on dry paper and photographed with a digital camera. Hematoma size was the sum of all lesion areas multiplied by slice thickness using Image J software (version 2.1, NIH).

### Hemoglobin assay

A colorimetric hemoglobin assay (Cayman Chemical, Ann Arbor, MI) was used to assess the hemoglobin contents of brains tissues. Briefly, mice were euthanized under deep anesthesia and infused by cold PBS (45 ml). The brains were removed immediately and divided into right and left hemispheres. Hemorrhagic hemispheres were isolated and washed in ice-cold PBS solution for three times. Then, tissues were homogenated with 300 μl distilled water. After being centrifuged at 10,000 g for 10 minutes, the supernatant was collected for determination of hemoglobin using the commercial kit according to the manufacturer’s instruction.

### Assay for oxidative stress

Oxidative stress was evaluated by determination of CuZu/Mn-superoxide dismutase (SOD), malondialdehyde (MDA), H_2_O_2_, O^2−^, and total antioxidant capacity (trolox equivalent antioxidant capacity, TEAC) with commercial kits as described previously^49, 50^. Briefly, mice were euthanized under deep anesthesia with chloral hydrate (500 mg/kg, intraperitoneal injection). The brains were removed immediately and divided into right and left hemispheres. The brain was cut into two parts and the hematoma in brain was well seen. Then, the brain hematoma area was dissected. After washing by PBS for three times, the peri-hemorrhagic brain tissues (<3 mm thickness) were isolated. Hematoma and peri-hematoma tissues were homogenated for assays with commercial kits. The commercial kits were purchased from Beyotime Institute of Biotechnology (Haimen, China). All assays were performed according to the manufacturer’s instruction.

### Quantitative Real-time-PCR (qRT-PCR)

Total RNA from the peri-hematoma tissues was isolated with TRIZOL (Invitrogen, Carlsbad, CA). The qRT-PCR was performed with a SYBR green PCR kit (Applied Biosystems) with ABI7500 Real-time PCR system (Applied Biosystems) as described previously^37, 51^. The primers are listed in Supplemental Table 1. Gene expression levels were quantified using a cDNA standard curve and data was normalized to β-actin, a housekeeping gene. Each reaction was performed in duplicate, and analysis was performed by the 2^−ΔΔCt^ method. Data is expressed as fold change.

### Immunohistochemistry staining

Immunohistochemistry staining was performed as described in our previous studies^14, 15^. Every primary antibody was tested before formal experiments for immunohistochemistry staining validation. Moreover, normal IgG was used for negative control to validate the specific staining in experiments. At 24 hours after cICH, mice were perfused under deep anesthesia with 20 ml cold PBS (pH = 7.4), followed by an infusion of 4% paraformaldehyde for 10 minutes. The brains were then removed and fixed in 4% paraformaldehyde at 4 °C overnight. The brains were dehydrated with 30% sucrose in formalin (pH = 7.4) and the frozen coronal slices (8 μm thick) were then sectioned in cryostat (CM3050S; Leica Microsystems, Bannockburn, IL). The sections were blocked in 8% normal goat serum for 4 hours, and incubated in specific primary antibodies overnight at 4 °C. After being washed three times by PBS, the sections were incubated with horseradish peroxidase-conjugated secondary antibodies. Staining is visualized using chromogenic substrate DAB. All the sections were counterstained by hematoxylin. Images were obtained with a digital microscope (Leica Microsystems, Berlin, Germany) and analyzed with a computerized image system with an analysis toolbox for immunohistochemistry image (Quantimet 500 Image Processing and Analysis System, Qwin V0200B software; Leica, Berlin, Germany).

The following antibodies were used for immunohistochemistry staining: NAD+ PH oxidase 1 (NOX-1, #ab78016, Abcam, Cambridge, MA, 1: 200 dilution), Iba-1 (#ab5076, Abcam, 1: 1,000 dilution), myeloperoxidase-1 (MPO-1, #BA0544, Boster, Wuhan, China, 1: 200 dilution), tumor necrosis factor–α (TNF-α, #ab6671, Abcam, 1: 200 dilution), interleukin-6 (IL-6, #ab9324, Abcam, 1: 500 dilution), intercellular adhesion molecule-1 (ICAM-1, #AF796, R&D Systems Co. Ltd., Minneapolis, MN, 1: 200 dilution) and vascular cell adhesion molecule-1 (VCAM-1, #AF643,RD system, 1: 200 dilution).

For quantification of immunohistochemistry staining, five sections per animal and five random microscope fields per section were chosen. The average number was then calculated.

### Protein extraction

For total protein extraction, brain tissues were washed in PBS (0.1 mmol/L), and homogenized with the buffer [Tris-HCl pH 7.5, 20 mmol/L; EDTA, 2 mmol/L; NP-40, 1%; Triton-100, 1%; PMSF, 2 mmol/L; leupeptin, 50 µg/ml; aprotinin, 25 µg/ml; pesptatin A, 10 ug/ml; and dithiothreitol (DTT), 2 mmol/L] with protein extraction reagent (Pierce) supplemented with a protease inhibitor cocktail (Pierce, Rockford, IL)^52^. For nuclear and cytosolic protein extraction, the brain tissues were washed in PBS and then performed with a commercial assay (Nuclear and Cytoplasmic Protein Extraction Kit, Beyotime, Haimen, China) according to the manufacturer’s instruction.

### Immunoblotting

The peri-hemorrhagic brain tissues were isolated for determination of protein expression of heme oxygenase 1 (HO-1) and nuclear factor-like 2 (Nrf2). Immunoblotting was performed in Odyssey Infrared Fluorescence Imaging System (Li-Cor) as described previously^37, 53, 54^. The protein concentration was determined by Bradford assay. About 30 µg samples were run on 10% SDS-PAGE. The proteins were electrotransferred to nitrocellulose membranes, probed with primary antibody (Nrf2, #ab31163, Abcam, 1: 3000; HO-1, #PB0050, Boster, 1: 2000) overnight, and then incubated with Infrared-Dyes-conjugated secondary antibodies (Li-Cor). The images were obtained with Odyssey Infrared Fluorescence Imaging System. All immunoblotting experiments were repeated at least three times.

### Measurements of NAD^+^ level

The assays for NAD^+^ level and SIRT1 activity were performed as described previously^13, 55^. NAD^+^ levels were determined with a NAD^+^ quantification kit (BioVision) according to instructions from the manufacturer.

### Statistical Analysis

Data were analyzed with GraphPad Prism-5 statistic software (La Jolla, CA). Values are presented as the mean ± SEM and analyzed by Student’s *t*-test, or ANOVA followed by Tukey post-hoc test, or repeated ANOVA (Fig. 6D and E). *P* < *0*.*05* was considered statistically significant.

## Electronic supplementary material

Supplemental material
