# Supplementary material for: Nicotinamide mononucleotide attenuates brain injury after intracerebral hemorrhage by activating Nrf2/HO-1 signaling pathway
Source: Sci Rep. 2017 Apr 6;7:717. doi: 10.1038/s41598-017-00851-z (PMC5429727; doi:10.1038/s41598-017-00851-z)
Supplement: Supplementary file 1 — Supplemental material [file 41598_2017_851_MOESM1_ESM.doc]

**Supplemental data**

**Nicotinamide mononucleotide attenuates brain injury after intracerebral hemorrhage by activating Nrf2/HO-1 signaling pathway**

**Running Head**: NMN treats intracerebral hemorrhage

**Chun-Chun Wei,^1*^ Yuan-Yuan Kong^1*^, Guo-Qiang Li^1*^, Yun-Feng Guan^1^, Pei Wang,^1,2#^ Chao-Yu Miao^1,3#^**

^1^Department of Pharmacology, Second Military Medical University, Shanghai, China

^2^Key Laboratory of Molecular Pharmacology and Drug Evaluation, Ministry of Education, Yantai University, Yantai, China

^3^Center of Stroke, Beijing Institute for Brain Disorders, Beijing, China

*These authors contributed equally to this work

^#^Correspondence to

Chao-Yu Miao, MD, PhD and Pei Wang, MD, PhD,

Department of Pharmacology, Second Military Medical University, Shanghai, China

E-mail: [cymiao@smmu.edu.cn](mailto:cymiao@smmu.edu.cn) and [pwang@smmu.edu.cn](mailto:pwang@smmu.edu.cn)

**Supplemental** Table 1. Primers for qRT-PCR

| Genes |  | DNA sequences (5’-3’) |
| --- | --- | --- |
| TNF-α | Forward | GGAACACGTCGTGGGATAATG |
|  | Reverse | GGCAGACTTTGGATGCTTCTT |
| IL-1β | Forward | GAAATGCCACCTTTTGACAGTG |
|  | Reverse | TGGATGCTCTCATCAGGACAG |
| IL-4 | Forward | GCTAT-TGATGGGTCTCACCC |
|  | Reverse | CAGGACGTCAAGGTA-CAGGA |
| IL-6 | Forward | CAAAGCCAGAGTCCTTCAGAG |
|  | Reverse | GCCACTCCTTCTGTGACTCC |
| IL-10 | Forward | GCCCTTTGCTATGGTGTCCTTTC |
|  | Reverse | TCCCTGGTTTCTCTTCCCAAGAC |
| β-actin | Forward | CGTTGACATCCGTAAAGACC |
|  | Reverse | AACAGTCCGCCTAGAAGCAC |

**Supplemental figure 1**


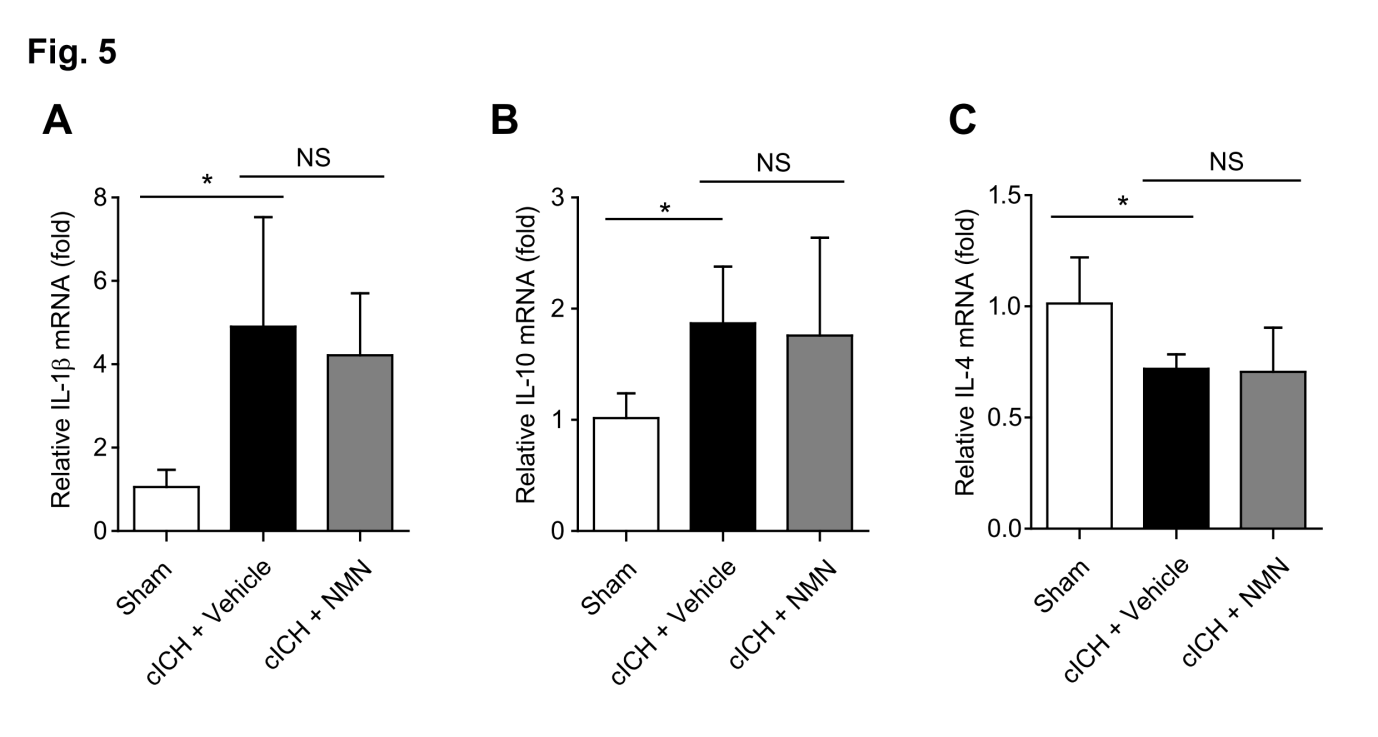


Effects of NMN on the mRNA expression of IL-1β, IL-10 and IL-4 in mouse cICH model. **P* < 0.05 vs Sham; NS, no significance. N = 6 per group.
